# *lac* Repressor Is an Antivirulence Factor of *Salmonella enterica*: Its Role in the Evolution of Virulence in *Salmonella*

**DOI:** 10.1371/journal.pone.0005789

**Published:** 2009-06-04

**Authors:** Sandeepa M. Eswarappa, Guruswamy Karnam, Arvindhan G. Nagarajan, Sangeeta Chakraborty, Dipshikha Chakravortty

**Affiliations:** Centre for Infectious Disease Research and Biosafety Laboratories, Department of Microbiology and Cell Biology, Indian Institute of Science, Bangalore, Karnataka, India; University of Hyderabad, India

## Abstract

The genus *Salmonella* includes many pathogens of great medical and veterinary importance. Bacteria belonging to this genus are very closely related to those belonging to the genus *Escherichia*. *lacZYA* operon and *lacI* are present in *Escherichia coli,* but not in *Salmonella enterica*. It has been proposed that *Salmonella* has lost *lacZYA* operon and *lacI* during evolution. In this study, we have investigated the physiological and evolutionary significance of the absence of *lacI* in *Salmonella enterica*. Using murine model of typhoid fever, we show that the expression of LacI causes a remarkable reduction in the virulence of *Salmonella enterica*. LacI also suppresses the ability of *Salmonella enterica* to proliferate inside murine macrophages. Microarray analysis revealed that LacI interferes with the expression of virulence genes of *Salmonella* pathogenicity island 2. This effect was confirmed by RT-PCR and Western blot analysis. Interestingly, we found that SBG0326 of *Salmonella bongori* is homologous to *lacI* of *Escherichia coli*. *Salmonella bongori* is the only other species of the genus *Salmonella* and it lacks the virulence genes of *Salmonella* pathogenicity island 2. Overall, our results demonstrate that LacI is an antivirulence factor of *Salmonella enterica* and suggest that absence of *lacI* has facilitated the acquisition of virulence genes of *Salmonella* pathogenicity island 2 in *Salmonella enterica* making it a successful systemic pathogen.

## Introduction

The genus *Salmonella* includes Gram-negative facultative intracellular pathogens that infect reptiles, birds and mammals. This genus includes two species, *viz*. *Salmonella bongori* (*S*. *bongori*) and *Salmonella enterica* (*S*. *enterica*). *S. enterica* is subdivided into many subspecies and serovars. In humans, *Salmonella enterica* serovar Typhi causes typhoid fever and serovars Typhimurium and Enteritidis cause food poisoning.

The virulence of *S*. *enterica* can be attributed largely to horizontally acquired genomic islands termed *Salmonella* pathogenicity islands (SPI) [1], [2]. Among them, *Salmonella* pathogenicity island 2 (SPI-2) imparts *S*. *enterica* an ability to cause systemic infection. SPI-2 is 40 kb in size and can be divided into two distinct parts. One part is 25 kb in size and encodes a type three secretion system (TTSS). This part is essential for the systemic virulence and is present in *S*. *enterica*, but absent in *S*. *bongori*. The other part is 15 kb in size and is not essential for the systemic virulence of *S*. *enterica*. This region encodes the tetrathionate reductase involved in anaerobic respiration and is present in both *S*. *bongori* and *S*. *enterica* [3]–[5]. Evolutionary significance of *S*. *bongori* not having the 25 kb part and *S*. *enterica* having both parts of SPI-2 is not clear.

Bacteria belonging to the genus *Salmonella* are closely related to those belonging to the genus *Escherichia* and they have diverged from a common ancestor about 100 million years ago [6]. Despite their close relationship, *E*. *coli* has more than 800 genes that are absent in the *S*. *enterica* genome and more than 1,100 *S*. *enterica* genes lack their homologues in *E*. *coli* [7]. The region containing *lacI* and the *lac* operon is one such locus and is present in *E*. *coli*, but absent in *S*. *enterica*. Thus, *E*. *coli* is a lactose fermenter, whereas *S*. *enterica* is a lactose non-fermenter. Nonetheless, diseases caused by lactose-fermenting *Salmonella* have been reported occasionally and they harbor genes responsible for lactose fermentation in extra-chromosomal genetic elements like plasmids [8]–[12].

The *lac* operon consists of three genes, *lacZ*, *lacY* and *lacA* which encode β-galactosidase, lactose permease and a transacetylase, respectively. *lacI* which encodes *lac* repressor (LacI) is located adjacent to *lacZYA,* but is transcribed as a separate message. LacI binds to the operator region of the *lac* operon and prevents the transcription of *lacZYA* genes unless lactose is present in the environment [13]. Expression of the *lac* operon when lactose is absent in the environment is costly for the bacteria [14] and LacI prevents such unnecessary expression of the *lac* operon. Constitutive expression of the *lac* operon lowers the fitness of the bacteria when lactose is not present in the environment [15], [16]. Therefore, LacI is very important to maintain the fitness of the bacterium that harbors the *lac* operon. None of the lactose-fermenting *Salmonella* strains (see above) are reported to harbor LacI, except *S*. Typhi strain ST-2 which harbors a mutant LacI with a diminished repressing ability [12].

It has been proposed that *S*. *enterica* has lost *lac* region (*lacI* and *lacZYA*) during evolution [17]. Many serovars of *S*. *enterica* are intestinal pathogens of mammals and like *E*. *coli* they are exposed to lactose in the mammalian gut. Then, why has *S*. *enterica* lost the ability to ferment lactose? So far, there are no studies which address this issue. In this study, we have investigated the physiological and evolutionary significance behind the loss of (or absence of) *lac* region in *S*. *enterica*. We have chosen LacI for this study because it is very important for a bacterium harboring the *lac* operon to have *lacI* as unnecessary expression of the *lac* operon lowers the fitness of the bacteria harboring it [15], [16]. Our study demonstrates that the expression of LacI in *S*. *enterica* reduces its virulence and suggests that lack of *lacI* has facilitated *S*. *enterica* to gain systemic virulence via SPI-2.

## Results

We used pTrc99A plasmid that harbors *lacI* gene to express LacI in *S*. *enterica* [18]. We deleted *lacI* from this plasmid to get pTrc(-LacI) plasmid and also we mobilized *lacI* along with its promoter from pTrc99A to pBR322 to get pBR322(+LacI). All these plasmids, *viz*. pTrc99A, pTrc(-LacI), pBR322 and pBR322(+LacI), were individually transformed into the wild-type (WT) strain of *S*. *enterica* serovar Typhimurium (*S*. Typhimurium). Expression of *lacI* in the strains harboring pTrc99A and pBR322(+LacI) was confirmed by RT-PCR and Western blot (Fig. S1 A and C). Growth of all these strains in Luria broth (LB) and M9 minimal medium were comparable to that of the parental WT strain (data not shown). Both pTrc99A and pBR322 have colE1 replicon and thus, have low to moderate (fifteen to twenty) copy numbers [19].

### *S*. *enterica* expressing LacI exhibits reduced virulence in murine typhoid fever model

**Figure 1:**
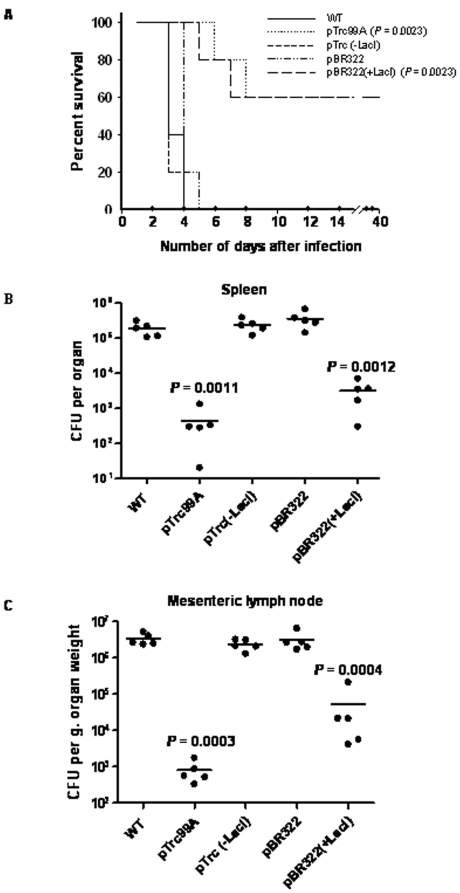
S. *enterica* expressing LacI shows attenuated virulence *in vivo*. (A) Survival plots of mice (n = 5 in each case) intra-peritoneally infected with *S*. *enterica* harboring different plasmids (10^4^ bacteria per mouse). Survival of mice infected with *S*. *enterica* expressing LacI [pTrc99A and pBR322(+LacI)] were compared to that of mice infected with the WT strain to calculate the statistical significance (*P* = 0.0023 for both the strains) using Logrank test. Bacterial load in (B) spleen and (C) mesenteric lymph node of mice (n = 5 in each case) intra-peritoneally infected (for 3 days) with *S*. *enterica* harboring different plasmids (10^3^ bacteria/mouse) are plotted in (B) and (C). The bacterial loads in organs of mice infected with experimental strains were compared to those of mice infected with the WT strain to calculate statistical significance by student's t-test. Horizontal lines in (B) and (C) indicate mean values. Graphs are representatives of at least two independent experiments.

Most of the known *S*. *enterica* serovars are pathogenic to one or the other vertebrate species. We were interested to know the effect of LacI on the virulence of *S*. *enterica*. We used murine typhoid fever model for this purpose. Mice were infected intra-peritoneally with *S*. *enterica* strains harboring different plasmids (see above) and infected mice were monitored for survival for 3 months. All the mice infected with the WT strain and strains harboring either pTrc(-LacI) or pBR322 (plasmids without *lacI*) were dead within 5 days of infection. Interestingly, 60% of the mice infected with strains harboring either pBR322(+LacI) or pTrc99A (plasmids expressing *lacI*) survived and did not succumb to the infection even after 3 months of infection (Fig. 1A).

Then, we examined the bacterial load in infected mice after 3 days of infection. We observed about 2 to 3 log order reduction in the bacterial load in spleen and mesenteric lymph node of the mice infected with strains harboring either pTrc99A or pBR322(+LacI) when compared to the organs of the mice infected with the WT strain (*P*<0.01). However, the bacterial load in spleen and mesenteric lymph node of the mice infected with strains harboring either pBR322 or pTrc(-LacI) were not different from those of the mice infected with the WT strain (*P*>0.05) (Fig. 1 B and C). These findings clearly show that LacI suppresses the virulence of *S*. *enterica*. LacI-dependent attenuation of *Salmonella* was also observed when the infection was done via intra-gastric route (Figure S2).

There is a possibility that plasmids may get lost from bacteria during infection and in such case, selecting bacteria on medium containing antibiotic will underestimate the bacterial load. To rule out such possibility, the organ homogenate was plated separately on medium with or without antibiotics. We observed that presence of antibiotic in the medium does not cause any change in the bacterial load estimation indicating that bacteria does not lose plasmid during infection.

**Figure 2:**
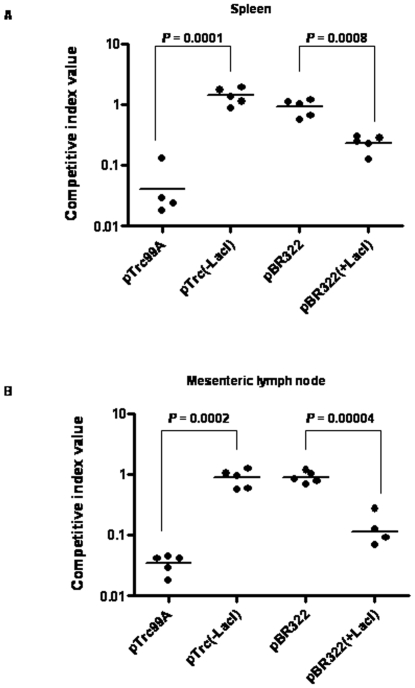
S. *enterica* expressing LacI are less competitive with respect to the WT strain. Mice (n = 5 in each case) were infected intra-peritoneally with a mixture of the WT strain and the indicated strain at 1∶1 ratio (total 10^3^ bacteria/mouse). After 3 days, CI values for all the strains were calculated with respect to the WT strain. CI values determined in spleen (A) and mesenteric lymph node (B) are plotted. Horizontal lines indicate mean values. Only four CI values are shown for the strain harboring pTrc99A in spleen and for the strain harboring pBR322(+LacI) in mesenteric lymph node as the fifth CI values were zero in both cases. Student's t-test was used to calculate the statistical significance. Graphs are representatives of two independent experiments.

To further confirm the effect of LacI on the virulence of *S*. *enterica*, we determined the competitive indices (CI) of strains expressing LacI with respect to the WT strain. Determination of CI is a very sensitive assay that compares the virulence of different strains [20]. For this assay, we used the WT strain constitutively expressing green fluorescent protein (GFP) to enable us to distinguish colonies of WT bacteria from the colonies of other strains on LB agar plate. The presence of GFP did not show any significant effect on the virulence of the WT strain (CI = 1.22±0.24; *P*>0.05). The CI values of strains harboring either pBR322(+LacI) or pTrc99A, determined in both spleen and mesenteric lymph node, were significantly (*P*<0.001) lesser than those of strains harboring either pBR322 or pTrc(-LacI) (Fig. 2). This result unequivocally demonstrates that LacI suppresses the virulence in *S*. *enterica*.

### *S*. *enterica* expressing LacI cannot proliferate inside murine macrophages

**Figure 3:**
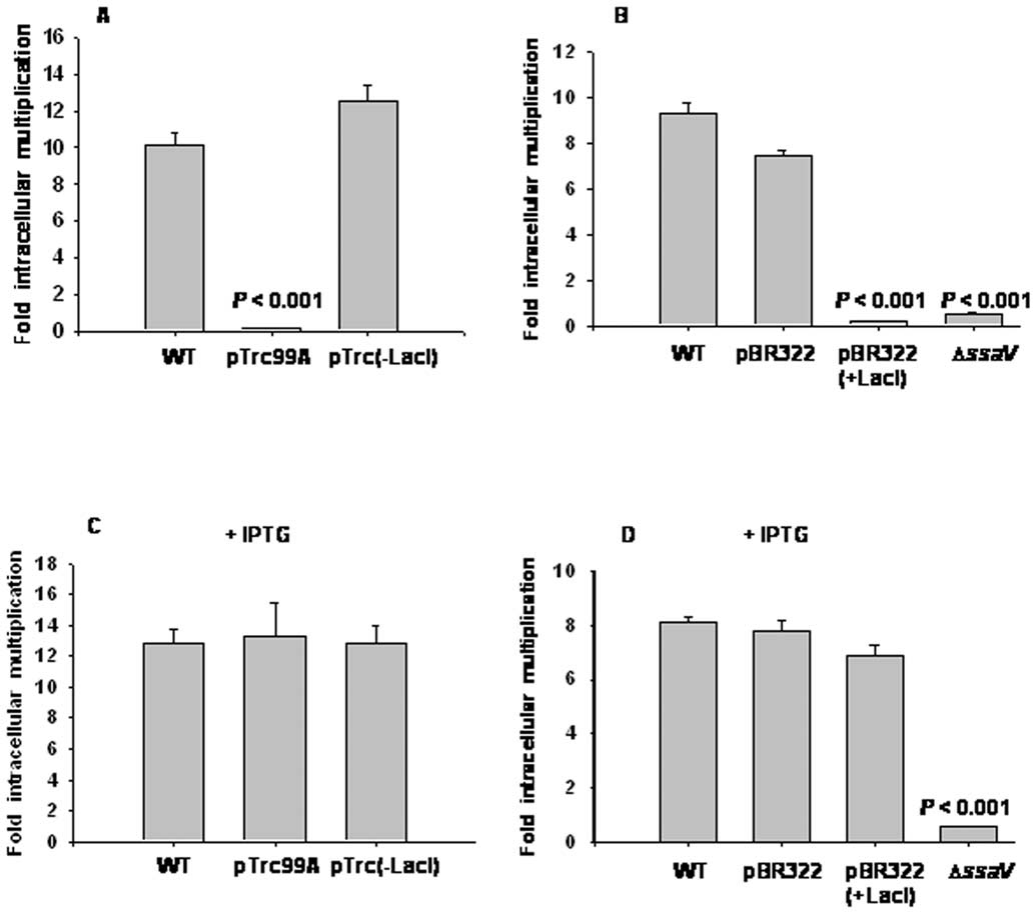
Expression of LacI in *S*. *enterica* suppresses its ability to proliferate inside murine macrophages. RAW-264.7 cells were infected with different strains as indicated at a MOI of 1∶1 in the absence (A and B) or presence (C and D) of 10 mM IPTG. In all experiments, fold multiplication of *S*. *enterica* inside RAW 264.7 cells was calculated by dividing the intracellular bacterial load at 16 h by the intracellular bacterial load at 2 h. The bars represent mean fold-multiplication±standard error. Fold-multiplication of different strains were compared with that of the WT strain to calculate the *P* value using Student's t-test. Graphs shown are representatives of at least two independent experiments done in triplicates in each case.

Our next aim was to understand how LacI is suppressing the virulence of *S*. *enterica*. To address this, we investigated the effect of LacI on the ability of *S*. *enterica* to multiply inside macrophages. Ability to multiply inside host macrophages is vital for the virulence of *S*. *enterica* [21]. We observed that strains harboring either pBR322(+LacI) or pTrc99A were unable to proliferate inside RAW 264.7 cells, a murine macrophage-like cell line. In contrast, proliferation of strains harboring either pTrc(-LacI) or pBR322 inside these macrophages were comparable to that of the WT strain (Fig. 3A and B). These finding illustrates that LacI suppresses the ability of *S*. *enterica* to multiply inside host macrophages and explains, at least in part, why *S*. *enterica* expressing LacI is less virulent. Nevertheless, LacI expression did not affect the entry of bacteria into epithelial (HeLa) cells as demonstrated by an invasion assay (Figure S3).

Our next aim was to investigate the mechanism of action of LacI that leads to the above observed effects in *S*. *enterica*; is DNA binding property of LacI responsible for these effects? To address this issue, we used isopropyl-*β*, D-thiogalactoside (IPTG). IPTG binds with high affinity to LacI and lowers its affinity for the operator DNA, the natural target of LacI [22]. However, IPTG does not affect the binding of LacI to non-operator DNA [23], [24]. Interestingly, LacI expressing strains [harboring either pTrc99A or pBR322(+LacI)] did not show any defect in their ability to multiply inside macrophages cultured in the presence of 10 mM IPTG. Proliferation of all the strains, *viz*. *S*. *enterica* having either pTrc99A or pTrc(-LacI) or pBR322 or pBR322(+LacI) were similar to that of the WT strain inside macrophages (Fig. 3C and D). Δ*ssaV*, a mutant strain defective in secretion by the SPI-2 type three secretion system (TTSS), was used as positive control because SPI-2 encoded TTSS is essential for *S*. *enterica* to proliferate inside host macrophages [5]. The phenotype of Δ*ssaV* strain was unaffected by IPTG (Fig. 3B and D).

This result clearly demonstrates that IPTG abolishes the inhibitory effect of LacI on the ability of *S*. *enterica* to multiply inside host macrophages. In addition, this result suggests that this inhibitory effect of LacI may be due to its binding to operator DNA and also it rules out the possible role of binding of LacI to a non-operator DNA sequence in this effect. This result also shows that LacI is indeed expressed in *S*. *enterica* harboring either pTrc99A or pBR322(+LacI) because the effect of IPTG is due to its binding with high affinity to LacI protein.

### Ability of LacI to bind to the operator sequence is not responsible for its effect on the virulence of *S. enterica*

**Figure 4:**
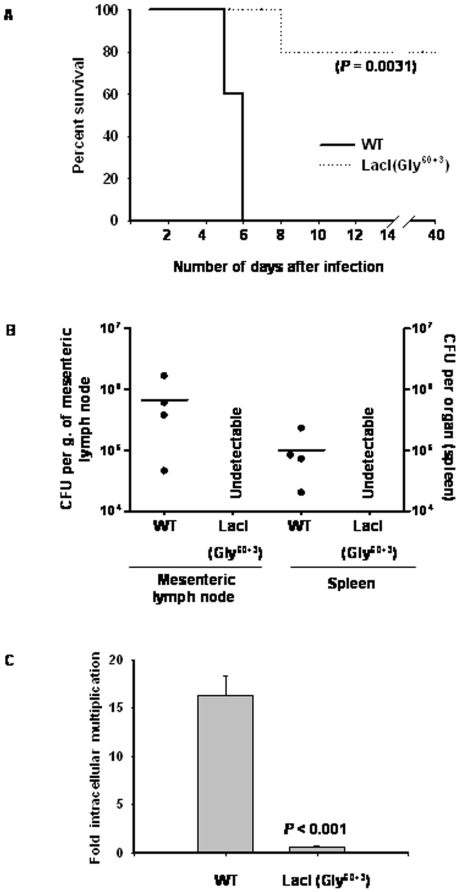
Ability of LacI to bind to the operator sequence is not responsible for its effect on the virulenceof *S. enterica*. (A) Survival plots of mice (n = 5 in each case) intra-peritoneally infected (10^4^ bacteria/mouse) with *S*. *enterica* expressing LacI(Gly^60+3^), a mutant of LacI that cannot bind to the operator sequence. Logrank test was used to calculate statistical significance. (B) Bacterial load in spleen and mesenteric lymph node of mice (n = 4 in each case) intra-peritoneally infected (for 3 days) with *S*. *enterica* expressing LacI(Gly^60+3^) (10^3^ bacteria/mouse). Horizontal lines indicate mean values. (C) Intracellular multiplication of *S*. *enterica* expressing LacI(Gly^60+3^) in RAW 264.7 cells. The bars represent mean fold-multiplication±standard error. Student's t-test was used to calculate *P* value. Graphs shown are representatives of at least two independent experiments.

In *E*. *coli*, LacI binds to the operator sequence present in the *lac* operon [25]. Mutating Gln^60^ to Gly and inserting three extra glycines after 60^th^ amino acid of LacI abolishes its ability to bind to the operator sequence without affecting its folding, assembly and inducer (IPTG) -binding property. However, the ability of this mutant LacI(Gly^60+3^) to bind to a non-operator DNA sequence is similar to that of the wild-type LacI [26]. We utilized this mutant LacI to investigate whether operator-binding property of LacI is responsible for its effect on the virulence of *S. enterica*. To our surprise, mice infected with *S*. *enterica* expressing LacI(Gly^60+3^) survived longer than those infected with the WT strain (Fig. 4A). Four out of five mice infected with *S*. *enterica* expressing LacI(Gly^60+3^) survived for more than 3 months after infection and did not succumb to infection. There were no detectable bacteria in the organs of mice infected with *S*. *enterica* expressing LacI(Gly^60+3^) after 3 days of infection (Fig. 4B). In addition, *S*. *enterica* expressing LacI(Gly^60+3^) could not proliferate inside macrophages (Fig. 4C). Our search for the presence of operator sequence in *S*. Typhimurium genome (gi: 16763390) using genomic BLAST program (BLASTN 2.2.18+) revealed that there is no sequence that is significantly similar (expect value<10) to the operator sequence (5′-tgttgtgtggaattgtgagcggataacaatttcacacagg-3′). These results clearly demonstrate that the effect of LacI on the virulence of *S*. *enterica* is independent of its ability to bind to the operator sequence.

### *S*. *enterica* expressing LacI is sensitive to mice serum

**Figure 5:**
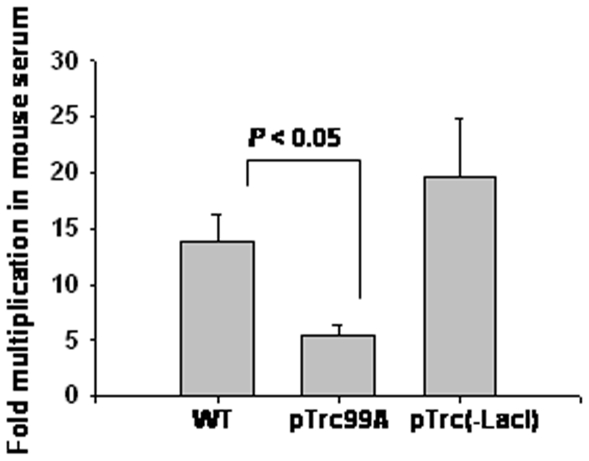
Sensitivity of *S*. *enterica* expressing LacI to mouse serum. Bacteria were grown in 96-well plates along with mouse serum as described in Materials and Methods for 4 h. Fold-replication was calculated by dividing the number of bacteria recovered after 4 h by the number of bacteria in the inoculum. Graph shown is a representative of three independent experiments done with triplicate samples.

Pathogenic bacteria like *S*. *enterica* that are capable of causing systemic disease are resistant to serum-mediated killing and this property is important for their virulence [27]. Interestingly, the growth of *S*. *enterica* harboring pTrc99A was significantly diminished in mouse serum (Fig. 5). *S*. *enterica* expressing LacI thus appears to be sensitive to serum-mediated killing; this might also contribute to the diminished virulence of *S*. *enterica* expressing LacI.

### LacI inhibits the expression of SPI-2 inside macrophages

Our next aim was to find out the possible targets of LacI in *S*. *enterica*. We used microarray technique for this purpose. Microarray expression profiling was done using RNA isolated from the WT strain and the strains harboring either pTrc99A or pTrc(-LacI) plasmids during mid-log phase growth in M9 minimal medium. The genes whose expression was affected in the strain harboring pTrc99A but not in the strain harboring pTrc(-LacI) were taken into consideration. We found 75 genes that were significantly down-regulated and 147 genes that were significantly up-regulated (>2-fold and *P*<0.02) in the strain harboring pTrc99A (Table S1 and Table S2). Among them, we looked for the genes that can influence the virulence of *S*. *enterica*. To our interest, 4 genes (*ssaK*, *ssaJ*, *ssrA* and orf245) belonging to SPI-2 and *phoP* were down-regulated in the strain harboring pTrc99A but not in the strain harboring pTrc(-LacI). Both SPI-2 and PhoP are essential for the virulence of *S*. *enterica* [3], [28]. This result encouraged us to look for the expression profile of these genes in the strain expressing LacI inside macrophages.

In addition to these genes, microarray analysis showed up-regulation of some flagella-related genes (*fliB*, *fliC* and *fliT*), genes involved in chemotaxis (*cheZ*, *cheY*, *cheB* and *cheW*) and genes belonging to SPI-1 (*sprB*, *prgK*, *spaP*, *spaO* and *invJ*). We did not pursue these genes further because up-regulation of these genes does not explain attenuation of virulence in *S*. *enterica*.

**Figure 6:**
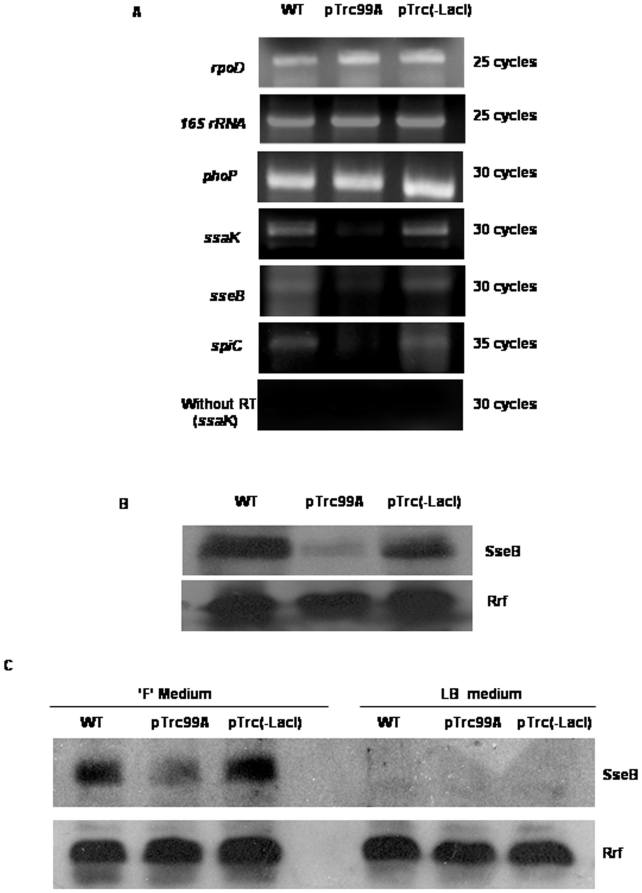
LacI interferes with SPI-2 expression. (A) The effect of LacI on the expression of virulence genes inside macrophages was analyzed by RT-PCR. cDNA was synthesized from the RNA isolated from intracellular bacteria and *ssaK*, *sseB*, *spiC* (representatives from SPI-2 island) and *phoP* were amplified by PCR. (B) Analysis of SseB expression by Western blot using total protein extracted from intracellular bacteria. (C) Analysis of SseB expression by Western blot using total protein extracted from bacteria grown in SPI-2-inducing (F medium) medium or LB for 2 h. Ribosomal recycling factor (Rrf) served as internal control. Results are representatives of at least two independent experiments.

The expression profile of SPI-2 genes in the strain harboring pTrc99A inside macrophages was investigated using RT-PCR. RNA was isolated from intracellular bacteria and subjected for reverse transcription followed by PCR. We chose three virulence genes, *viz*. *ssaK*, *sseB* and *spiC* which represent three different operons of the 25 kb part of SPI-2 that encodes a TTSS. Expression of *ssaK*, *sseB* and *spiC* was reduced in the strain harboring pTrc99A inside macrophages. This reduction was not observed in the strain harboring pTrc(-LacI) indicating that LacI interferes with the expression of these genes. However, the expression of *phoP* was unaffected (Fig. 6A). These results were confirmed using quantitative real-time PCR analysis (Fig. S4).

To further confirm the effect of LacI on SPI-2 expression, we looked at SseB protein level in intracellular bacteria recovered from macrophages by Western blotting. We observed a remarkable reduction in SseB protein level in the strain harboring pTrc99A but not in the strain harboring pTrc(-LacI) inside macrophages (Fig. 6B). Expression of SPI-2 can be induced *in vitro* by using F medium [29]. This medium mimics intra-phagosomal environment as it is low in magnesium and phosphorous and acidic in pH. When grown in F medium for 2 h, there was significant decrease in the SseB protein level in the strain harboring pTrc99A but not in the strain harboring pTrc(-LacI) (Fig. 6C).

These results unequivocally demonstrate that LacI interferes with the expression of SPI-2 genes inside macrophages explaining, at least in part, why *S*. *enterica* expressing LacI are unable to proliferate inside macrophages and are attenuated in virulence *in vivo*. As some of the SPI-2 mutants are shown to be sensitive to serum mediated killing [30], this result also explains the serum sensitivity of *S*. *enterica* expressing LacI.

### SBG0326 of *S*. *bongori* is homologous to *lacI*

**Figure 7:**
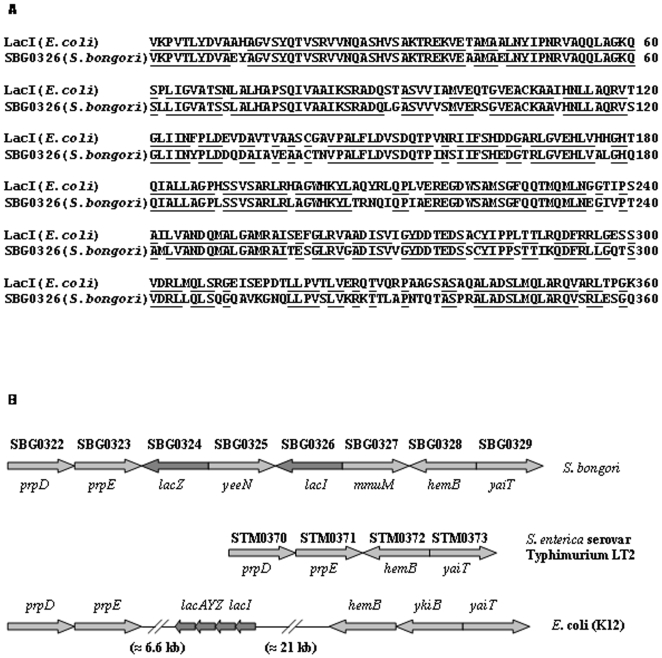
SBG0326 of *S*. *bongori* is homologous to *lacI* of *E*. *coli*. (A) Alignment of predicted amino acid sequences of SBG0326 and LacI (*E*. *coli* K12). Both of them share 77% identity and 88% similarity. Identical residues are underlined. (C) Schematic diagram showing organization of *prpE* to *hemB* locus in *S*. *bongori*, *S*. Typhimurium and *E*. *coli* (K12). Each arrow represents indicated gene. Genes belonging to *lac* region are shown in darker shade. In case of *S*. *bongori* and *S*. Typhimurium, the names of the homologous genes in *E. coli* are given below the arrows. Arrows are not to scale.

*S. bongori*, the only other *Salmonella* species, is found as a commensal of cold-blooded animals and it cannot cause systemic infections in mammals. 25 kb region of SPI-2 that encodes TTSS is absent in *S*. *bongori*, but is present in *S*. *enterica* [5]. This region is essential for the systemic virulence of *S*. *enterica*. These facts prompted us to look for the presence of *lacI* homologue/s in *S*. *bongori* genome. Interestingly, we observed that SBG0326 of *S*. *bongori* is homologous to *lacI* of *E*. *coli*. The predicted amino acid sequences of protein encoded by SBG0326 and LacI share 77% identity and 88% similarity (Fig. 7A). However, we did not find any transcript corresponding to *lacI* in *S*. *bongori* suggesting that SBG0326 is probably a pseudogene (Fig. S1 A and B).

Our search also revealed that SBG0324 of *S*. *bongori* is homologous to *lacZ* of *E*. *coli*. The predicted amino acid sequences of protein encoded by SBG0324 and LacZ share 79% identity and 87% similarity. There were no genes that are homologous to *lacY* and *lacA* in *S*. *bongori* genome.

We observed that *prpE* and *hemB* are contiguous in *S*. *enterica*. However, these two genes are separated by four genes in *S*. *bongori* two of which are SBG0324 (*lacZ*) and SBG0326 (*lacI*). In *E*. *coli*, *prpE* and *hemB* are separated by 34.161 kb region that includes *lac* operon and *lacI* (Fig. 7B). These observations suggest that *S*. *bongori* has partially lost the *lac* region, whereas *S*. *enterica* has lost it completely.

## Discussion

The evolution of bacterial pathogens from their nonpathogenic ancestors is an exciting saga which involves many kinds of fascinating genetic events. This is central to the understanding of both the origin of infectious diseases and the emergence of new pathogens. Some bacteria have horizontally acquired genetic elements encoding virulence factors. For example, *Salmonella* has many horizontally acquired pathogenicity islands [1]. In some cases, virulence results from allelic differences in homologous genes. For example, genetic variation of *fimH*, a gene that encode adhesin of type 1 fimbriae, is observed in nonpathogenic and uropathogenic *E*. *coli* [31]. Some bacteria have selectively inactivated genes that are no longer compatible with the pathogenic lifestyle of the bacteria either by point mutation, insertion, or deletion. Such genes are called ‘antivirulence genes’ [32]. For example, *cadA* and *ompT* are absent in *Shigella,* but are present in nonpathogenic *E*. *coli*. Introduction of either *cadA* or *ompT* attenuates virulence in *Shigella* [33], [34]. *zirTS*, *grvA* and *pcgL* are the antivirulence genes reported in *S*. *enterica* [35]–[37]. However, all these genes are present in *S*. *enterica* and modulate its virulence. So far, there is no report of an antivirulence gene of *S*. *enterica* that has been lost during evolution. Our study unequivocally demonstrates that *lacI* is an antivirulence gene of *S*. *enterica* which has been lost from it, but present in closely related *S*. *bongori* and *E*. *coli*.

We recognize the fact that the observed remarkable inhibitory effect of LacI on the virulence of *S*. *enterica* may be an exaggerated effect of LacI expression using plasmids having low to moderate copy numbers (fifteen to twenty). pTrc99A plasmid has operator region that can quench a part of LacI which reduces the amount of free LacI and decreases the effect of copy number of the plasmid on freely available LacI. Nevertheless, a slight inhibitory effect on the expression of virulence gene is sufficient to drive a genetic trait (if dispensable) towards negative selection.

Plasmids lacking LacI [pTrc99(-LacI) and pBR322] express beta-lactamase and tetracycline resistance proteins which are foreign to *Salmonella*, but they do not affect the virulence of *S*. *enterica*. Moreover, constitutive expression of green fluorescent protein also did not affect the virulence of *S*. *enterica*. Therefore, the effect of LacI on the virulence of *S*. *enterica* is unlikely to be just an artifact caused by the expression of a foreign protein.

There are two possibilities by which LacI can inhibit the virulence of *S*. *enterica*: either by binding to DNA or by binding to a protein. With respect to DNA binding, IPTG and Gly^60+3^ mutation in LacI have same effect on the function of LacI. Both of them inhibit the LacI binding to the operator DNA without affecting its binding to non-operator sequence. IPTG could reverse the inhibitory effect of LacI on the ability of *S*. *enterica* to proliferate inside macrophages, whereas *S*. *enterica* expressing LacI mutant (Gly^60+3^) was unable to proliferate inside macrophages. These results indicate that the effect of LacI on the virulence of *S*. *enterica* is not because of its DNA binding property. The other possibility is the binding of LacI to a protein. As LacI can form homodimer and homotetramer, there is a possibility of LacI forming heterodimer or heterotetramer with other transcriptional factors or any proteins present in *S*. *enterica* and IPTG might inhibit such protein-protein interactions. All these possibilities need to be investigated.

Our study demonstrates that the antivirulence property of LacI in *S*. *enterica* is, at least in part, because of its interference with the expression of SPI-2. Acquisition of SPI-2 by *S*. *enterica* is a landmark event in the evolution of *Salmonella*. SPI-2 has enabled *S*. *enterica* to survive and multiply inside macrophages resisting their antimicrobial activities [38], [39]. This ability is essential for *S*. *enterica* to cause systemic infection [3], [5]. Thus, acquisition of virulence genes of SPI-2 has made it possible for *S*. *enterica* to extend its niche beyond intestine, whereas absence of the same has made *S*. *bongori* a mere commensal of cold-blooded animals. Based on these facts and our results, we propose that presence of SBG0326 (*lacI*) has probably hindered the acquisition of virulence genes of SPI-2 in *S*. *bongori*, whereas absence of *lacI* has facilitated the same in *S*. *enterica* making it a successful systemic pathogen. Thus, *lacI* has played a remarkable role in the evolution of virulence in *Salmonella*.

## Materials and Methods

### Bacterial strains, plasmids and culture conditions

*Salmonella enterica* serovar Typhimurium strain NCTC 12023 was used as wild-type (WT) in all experiments. This was the parental strain for all other strains. Δ*ssaV* and *S*. *bongori* IH4 strains were kind gifts from Prof. Michael Hensel. Various plasmids used in this study are listed in Table S3. pTrc(-LacI) was constructed by digesting pTrc99A plasmid with BssHII to remove 872 nucleotide region encoding LacI and self-ligating the remaining plasmid. The deleted region included the promoter of *lacI*. *lacI,* including its promoter region, of pTrc99A was mobilized into EcoRI and HindIII sites of pBR322 to get pBR322(+LacI) plasmid. Primers used for constructing different plasmids are listed in Table S4. All plasmids were individually transformed to WT *S*. *enterica*. Bacteria were routinely cultured in Luria broth (LB) at 37°C. Optical density of the bacterial culture at 600 nm was measured every hour till stationary phase reached in order to monitor the growth of all the strains in LB or M9 minimal medium.

### Mice infections

All procedures with animals were carried out in accordance with institutionally approved protocols. Six- to eight-week-old BALB/c mice were infected intra-peritoneally with *S*. *enterica* in all the experiments. A dose of 10^3^ bacteria per mouse was used to investigate the bacterial load in spleen and mesenteric lymph node. After 3 days of infection, spleen and mesenteric lymph node of infected mice were harvested and homogenized. Bacteria were enumerated after plating a dilution series onto LB agar. A dose of 10^4^ bacteria per mouse was used to follow the survival of the infected mice for 3 months. To calculate the competitive indices (CI) of *S*. *enterica* harboring different plasmids, mice were infected with equal amounts of WT *S*. *enterica* constitutively expressing green fluorescent protein (pFPV25.1) and the experimental strain (a ratio of 1∶1). A total of 10^3^ bacteria per mouse were inoculated. After 3 days, spleen and mesenteric lymph node were harvested, homogenized and the homogenate was plated onto LB agar. Bacterial colonies with (WT strain) or without (experimental strains) green fluorescence were enumerated. The CI was defined as the ratio of the experimental strain to the WT strain recovered from the mice divided by their ratio in the initial inoculum [20]. Presence of green fluorescent protein did not affect the virulence of *S*. *enterica*.

### Serum sensitivity assay

Blood collected by retro-orbital bleeding of mice was used to extract the serum. Healthy, Six- to eight-week-old BALB/c mice (that were to be sacrificed for some other purpose) were used for retro-orbital bleeding. Bacteria in exponential phase were seeded in a 96-well plate at a concentration of 2 to 5×10^4^ bacteria per well in 50 µl of a solution containing 0.5% tryptone and 0.5% sodium chloride. 50 µl of mouse serum was added to each well and incubated at 37°C in shaking conditions for 4 h. The samples were then plated at different dilutions on LB plates. Fold multiplication was calculated by dividing the number of bacteria recovered after 4 h by the number of bacteria in the inoculum.

### Eukaryotic cell culture and intracellular proliferation assays

RAW 264.7 cells were grown routinely in Dulbecco's Modified Eagle's Medium (DMEM; Sigma-Aldrich, USA) supplemented with 10% fetal calf serum (Sigma-Aldrich, USA) at 37°C in 5% CO_2_. RAW 264.7 cells (3×10^5^ per well) were seeded in 24-well plates 12 to 24 h prior to infection. These cells were infected with overnight culture of a specific strain of *S*. *enterica* as described earlier at a multiplicity of infection (MOI) of 1∶1 [40]. To check the expression of SPI-2 genes by RT-PCR and western blot, an MOI of 50∶1 was used. Fold intracellular multiplication was calculated by dividing the intracellular bacterial load at 16 h by the intracellular bacterial load at 2 h.

### Microarray analysis

The WT strain and the strains harboring either pTrc99A or pTrc(-LacI) were grown in M9 minimal medium for 9 h (mid-log phase). After 9 h, total RNA was isolated from equal number of bacteria of each strain using ‘RNeasy Mini Kit’ (QIAGEN, USA). ‘RNAprotect Bacteria Reagent’ (QIAGEN, USA) was used to stabilize RNA before bacterial cells were lysed. The quality of the RNA was checked using ‘Agilent - 2100 Bioanalyzer’ (Agilent Technologies, USA). RNA was amplified using ‘MessageAmp™ II-Bacteria RNA Amplification kit’ (Ambion, USA) and labeled using ‘Low Input RNA amplification kit’ (Agilent Technologies, USA). Hybridization was done using ‘In situ Hybridization kit’ (Agilent Technologies, USA). Dye-swap was included in the hybridization plan. Hybridization plan was as follows: WT_Cy3_ *vs*. pTrc99A_Cy5_, WT_Cy5_ *vs*. pTrc99A_Cy3_, WT_Cy3_ *vs*. pTrc(-LacI)_Cy5_, WT_Cy5_ *vs*. pTrc(-LacI)_Cy3_. Slides were scanned at 5 µ resolution using Agilent scanner. Automated feature extraction was done using Agilent's Feature Extraction Software. Analysis of feature extracted data was done using Agilent's GeneSpring GX V 7.3.1 software. The normalization was done using GeneSpring GX using the recommended Per Chip and Per Gene Data Transformation. Genes with ratio of 2 and above were considered as up-regulated and with ratio 0.5 and below were considered down-regulated. *P* value was calculated by GeneSpring GX for each gene on the basis of replicate probes to indicate statistical significance. *P* value less than 0.02 was taken as statistically significant. Raw data of microarray experiment is provided in Table S5. All microarray data reported in the manuscript is described in accordance with MIAME guidelines and the data from the experiments are deposited in GEO (accesssion no. GSE15950 and GPL8520).

### Extraction of bacterial RNA and protein from infected cells

Twelve hours after infection, RAW 264.7 cells were lysed on ice by incubating for 30 min in 0.1% SDS, 1% acidic phenol, and 19% ethanol in water. Bacteria were isolated from the lysate by centrifugation; RNA and total protein were extracted from the pellet using TRI reagent (Sigma-Aldrich, USA) according to the manufacturer's protocol. In each case, bacteria were recovered from two 6-well plates of infected RAW 264.7 cells and infected cells were pooled to isolate RNA and protein.

### RT-PCR

RNA was extracted from an overnight bacterial culture to check the expression of *lacI* and from intracellular bacteria (isolated from RAW 264.7 cells) to check the expression of SPI-2 genes. RNA (2 µg) was treated with RNase-free DNase (Fermentas, USA) and then reverse transcribed using a reverse transcription system (Promega, USA). The cDNA thus generated was amplified using specific primers designed for *lacI*, *phoP*, *ssaK*, *sseB*, *spiC*, *rpoD* and *16S rRNA*. The nucleotide sequences of all primers are given in Table S4.

### Western blot

To check the expression of SPI-2, total protein was extracted from intracellular bacteria (isolated from RAW 264.7 cells) and also from bacteria grown in F or LB medium for 2 h. The F medium was prepared as described previously [29]. Protein was electrophoresed and transferred to a polyvinylidene difluoride membrane (Millipore, USA). The membrane was then probed with SseB or Rrf antibody (a kind gift from Prof. Hensel and Prof. Varshney, respectively) and respective horse radish peroxidase- (HRP) conjugated secondary antibody (Bangalore Genei, India). Immune complexes were detected using an enhanced chemiluminescence reagent (PerkinElmer, USA).

### Bioinformatic analysis

The complete genome sequence of *S*. *bongori* 12419 ATCC 43975 available in Welcome Trust Sanger Institute website (http://www.sanger.ac.uk/Projects/Salmonella/) and the nucleotide sequences of *lacI* and *lacZYA* genes of *E*. *coli* K12 were used to search for the presence of *lac* region in *S*. *bongori*; ‘BLAST 2 sequences’ tool was used for this analysis [41]. ExPASy-Translate tool was used to predict amino acid sequences [42]. ClustalW program was used for amino acid sequence alignment [43].

### Statistical methods

Logrank test was used to analyze the survival curves of mice infected with different strains of *S*. *enterica*. Student's t-test was used to analyze the bacterial load in the organs of infected mice and also to analyze the ability of different strains of *S*. *enterica* to multiply inside macrophages and in the presence of mouse serum.

## Supporting Information

Table S1 List of genes which are down-regulated in the strain having pTrc99A but not in the strain having pTrc(-LacI)
(0.16 MB DOC)

Table S2 List of genes which are up-regulated in the strain having pTrc99A but not in the strain having pTrc(-LacI)
(0.30 MB DOC)

Table S3 Plasmid constructs used in this study
(0.03 MB DOC)

Table S4 Primers used in this study
(0.03 MB DOC)

Table S5 Raw data of microarray experiment
(8.59 MB XLS)

Figure S1 Analysis of expression of LacI. (A) RT-PCR analysis of lacI expression in different strains of Salmonella and E. coli. RNA isolated from overnight culture of bacteria was treated with RNase-free DNase and reverse transcribed. cDNA thus generated was used for PCR amplification. (B) PCR amplification of lacI from the genomic DNA of bacteria. In both (A) and (B), PCR was done using lacI-specific primers. rpoD was used as internal control. (C) Western blot analysis of LacI expression in different strains of Salmonella. Equal number of bacteria from overnight culture was lysed and the lysate was used for Western blot analysis. LacI antibody was from Abcam.
(1.24 MB TIF)

Figure S2 Effect of LacI on the proliferation of Salmonella in mice infected via intra-gastric route. The graph shows the bacterial load of the spleens of mice infected with Salmonella.
(8.43 MB TIF)

Figure S3 Expression of LacI in Salmonella does not affect invasion. HeLa cells were infected as described in Materials and Methods with log-phase bacteria with an MOI of 1. Cells were lysed at 1 h after infection to recover the intracellular bacteria. Percentage of entry was calculated by comparing the number of entered bacteria with the number of bacteria in the pre-inoculum.
(8.43 MB TIF)

Figure S4 LacI interferes with SPI-2 expression inside macrophages. The effect of LacI on the expression of virulence genes was analyzed by quantitative real-time PCR. The cDNA was synthesized from the RNA isolated from intracellular bacteria and was used to perform quantitative real-time PCR using DyNAmo™ HS SYBR® Green qPCR Kit. Following conditions were used for all reactions: 40 cycles of 94°C for 20 sec, 57°C for 30 sec and 72°C for 30 sec. Relative amounts of ssaK, sseB and phoP mRNA with respect to rpoD mRNA was quantified.
(8.43 MB TIF)
