# Supplementary material for: lac Repressor Is an Antivirulence Factor of Salmonella enterica: Its Role in the Evolution of Virulence in Salmonella
Source: PLoS One. 2009 Jun 4;4(6):e5789. doi: 10.1371/journal.pone.0005789 (PMC2686271; doi:10.1371/journal.pone.0005789)
Supplement: Table S1 — List of genes which are down-regulated in the strain having pTrc99A but not in the strain having pTrc(-LacI) (0.16 MB DOC) [file pone.0005789.s001.doc]

| **Gene** | **Ratio_pTrc99A (cy5) vs. WT (cy3)** | ***P*-value**  **Table S1: List of genes which are down-regulated in the strain having pTrc99A but not in the strain having pTrc(-LacI)** | **Ratio_pTrc99A (cy3) vs. WT (cy5)** | ***P*-value** | **Description** | **accessions** |
| --- | --- | --- | --- | --- | --- | --- |
| PSLT049 | 0.11054612 | 0.017094163 | 0.4644519 | 0.013207458 | putative DNA polymerase III epsilon subunit | tigr|PSLT049 |
| PSLT072 | 0.23245513 | 0.007779599 | 0.45983914 | 0.003620555 | putative transglycosylase (finP) | tigr|PSLT072 |
| STM0312 | 0.34187812 | 0.012150628 | 0.10001769 | 0.0124934 | putative periplasmic protein (yafK) | tigr|STM0312 |
| STM0313 | 0.1859199 | 0.009263713 | 0.32636902 | 0.00615291 | DNA polymerase IV, devoid of proofreading, damage-inducible (dinP) | tigr|STM0313 |
| STM0378 | 0.48774272 | 0.003404498 | 0.32027215 | 9.01E-04 | putative inner membrane protein (yaiY) | tigr|STM0378 |
| STM0469 | 0.24094176 | 0.005751506 | 0.2594208 | 0.005834831 | putative 50S ribosomal protein L31 (second copy) (rpmE2) | tigr|STM0469 |
| STM0470 | 0.38123122 | 0.003092454 | 0.37155506 | 6.79E-04 | putative 50S ribosomal protein L36 (second copy) (rpmJ2) | tigr|STM0470 |
| STM0586 | 0.27122167 | 0.017412867 | 0.4310352 | 0.012056172 | enterochelin esterase (fes) | tigr|STM0586 |
| STM0756 | 0.28724927 | 0.005743109 | 0.28907168 | 0.001745683 | quinolinate synthetase, A protein (nadA) | tigr|STM0756 |
| STM1128 | 0.15814397 | 0.018135061 | 0.2774961 | 0.005190163 | putative sodium/glucose cotransporter | tigr|STM1128 |
| STM1167 | 0.117692985 | 0.017268877 | 0.4128455 | 6.14E-04 | acetylation of N-terminal alanine of 30S ribosomal (rimJ) | tigr|STM1167 |
| STM1168 | 0.10802454 | 0.007040702 | 0.30639127 | 1.21E-04 | putative cytoplasmic protein (yceH) | tigr|STM1168 |
| STM1174 | 0.43565693 | 0.011296853 | 0.4745117 | 0.007098698 | flagellar biosynthesis, cell-proximal portion of basal-body rod (flgB) | tigr|STM1174 |
| STM1187 | 0.36811608 | 0.003418629 | 0.20407522 | 0.001242941 | 23S rRNA pseudouridylate synthase (rluC) | tigr|STM1187 |
| STM1193 | 0.27421016 | 0.007144735 | 0.48499736 | 0.019466326 | 3-oxoacyl-[acyl-carrier-protein] synthase III acetylCoA ACP transacylase (fabH) | tigr|STM1193 |
| STM1194 | 0.19092406 | 0.019846488 | 0.3088231 | 9.73E-04 | malonyl-CoA-[acyl-carrier-protein] transacylase (fabD) | tigr|STM1194 |
| STM1197 | 0.24670605 | 0.014111713 | 0.34952793 | 0.004510033 | 3-oxoacyl-[acyl-carrier-protein] synthase II (fabF) | tigr|STM1197 |
| STM1201 | 0.38261154 | 0.003134371 | 0.46097296 | 1.33E-04 | DNA polymerase III, delta prime subunit (holB) | tigr|STM1201 |
| STM1206 | 0.26258424 | 0.001552325 | 0.2874256 | 1.25E-04 | putative outer membrane lipoprotein (ycfL) | tigr|STM1206 |
| STM1213 | 0.1976371 | 0.002411711 | 0.276295 | 0.010226557 | putative transcriptional repressor (TetR/AcrR family) (ycfQ) | tigr|STM1213 |
| STM1221 | 0.341318 | 0.001068382 | 0.41898808 | 5.12E-04 | putative nicotinate-nucleotide dimethylbenzimidazolephosphoribosltransferase, homolog of virulence factor (cobB) | tigr|STM1221 |
| STM1222 | 0.46277773 | 0.012724593 | 0.34613067 | 0.010351832 | ABC superfamily (peri_perm), spermidine/putrescine transporter (potD) | tigr|STM1222 |
| STM1227 | 0.46888888 | 0.010104727 | 0.1184153 | 0.001351676 | putative peptidase T(aminotripeptidase) (pepT) | tigr|STM1227 |
| STM1231 | 0.237338 | 0.017673649 | 0.2397284 | 0.004784505 | response regulator in two-component regulatory system with (phoP) | tigr|STM1231 |
| STM1237 | 0.1793446 | 0.002483874 | 0.15183714 | 7.38E-05 | putative ribosomal large subunit pseudouridine synthase (ymfC) | tigr|STM1237 |
| STM1239 | 0.13073464 | 0.01999164 | 0.19663723 | 0.01132283 | putative cytoplasmic protein | tigr|STM1239 |
| STM1242 | 0.14327161 | 0.005653306 | 0.11462897 | 0.005038829 | putative envelope protein (envE) | tigr|STM1242 |
| STM1249 | 0.24959287 | 0.018685507 | 0.29025167 | 0.01165824 | putative periplasmic protein | tigr|STM1249 |
| STM1250 | 0.23916973 | 0.007045298 | 0.44739595 | 0.011478215 | putative cytoplasmic protein | tigr|STM1250 |
| STM1253 | 0.20528804 | 0.007830328 | 0.31488422 | 0.0181183 | putative inner membrane protein | tigr|STM1253 |
| STM1264 | 0.4651844 | 0.002885787 | 0.47333425 | 7.58E-04 | Aminoglycoside adenyltransferase (aadA) | tigr|STM1264 |
| STM1267 | 0.13078405 | 0.019695243 | 0.19588144 | 0.005002303 | putative cytoplasmic protein | tigr|STM1267 |
| STM1288 | 0.1709956 | 0.009437072 | 0.31505066 | 0.001541154 | putative aldehyde reductase | tigr|STM1288 |
| STM1308 | 0.27389154 | 0.007379448 | 0.46344444 | 0.01401941 | periplasmic protein related to spheroblast formation (spy) | tigr|STM1308 |
| STM1311 | 0.11943024 | 0.005124717 | 0.31246206 | 0.001272174 | transcriptional activator of ntrL gene (osmE) | tigr|STM1311 |
| STM1323 | 0.2500761 | 0.006629454 | 0.42089772 | 8.70E-05 | putative regulator (yniB) | tigr|STM1323 |
| STM1326 | 0.25687772 | 0.001622309 | 0.45333582 | 6.11E-04 | 6-phosphofructokinase II (pfkB) | tigr|STM1326 |
| STM1337 | 0.17396572 | 0.009643821 | 0.46568742 | 0.008065008 | phenylalanine tRNA synthetase, alpha-subunit (pheS) | tigr|STM1337 |
| STM1339 | 0.2886507 | 0.011402715 | 0.47054258 | 2.90E-04 | integration host factor (IHF), alpha subunit (himA) | tigr|STM1339 |
| STM1340 | 0.2901486 | 0.007006723 | 0.3323512 | 0.012357634 | ABC superfamily (membrane), vitamin B12 transport protein (btuC) | tigr|STM1340 |
| STM1342 | 0.20999435 | 0.006218195 | 0.47368297 | 0.003079088 | ABC superfamily (binding protein), vitamin B12 transport (btuD) | tigr|STM1342 |
| STM1344 | 0.22711599 | 0.001860082 | 0.38972932 | 0.004195568 | putative Diguanylate cyclase/phosphodiesterase domain 1 (ydiV) | tigr|STM1344 |
| STM1370 | 0.3217257 | 0.001382212 | 0.4700702 | 0.003064791 | putative ABC transporter (sufB) | tigr|STM1370 |
| STM1381 | 0.05859216 | 0.014587139 | 0.43131617 | 0.006931345 | putative cytoplasmic protein (orf245) | tigr|STM1381 |
| STM1392 | 0.22888577 | 0.012623766 | 0.39509386 | 0.001111093 | Secretion system regulator:Sensor component (ssrA) | tigr|STM1392 |
| STM1409 | 0.07934218 | 0.010164264 | 0.44820517 | 0.008351254 | Secretion system apparatus: homology with the yscJ/mxiJ/prgK (ssaJ) | tigr|STM1409 |
| STM1411 | 0.08745591 | 0.011770941 | 0.42208916 | 0.013636406 | Secretion system apparatus (ssaK) | tigr|STM1411 |
| STM1425 | 0.21581365 | 0.011326371 | 0.29468453 | 0.019057091 | putative MATE family transport protein (ydhE) | tigr|STM1425 |
| STM1447 | 0.32215843 | 0.003068822 | 0.35045195 | 0.003094286 | putative outer membrane lipoprotein (ydhA) | tigr|STM1447 |
| STM1564 | 0.11218579 | 0.006463698 | 0.14428504 | 6.19E-05 | putative cytoplasmic protein (yddX) | tigr|STM1564 |
| STM1689 | 0.1244178 | 0.005060711 | 0.49911702 | 0.00503282 | phage shock protein regulatory gene, activates expression (pspB) | tigr|STM1689 |
| STM1705 | 0.30085775 | 0.005788756 | 0.19148134 | 2.27E-04 | osmotically inducible lipoprotein (osmB) | tigr|STM1705 |
| STM1830 | 0.4288986 | 0.004332804 | 0.37400335 | 2.45E-04 | Sugar Specific PTS family, mannose-specific enzyme IIAB (manX) | tigr|STM1830 |
| STM1998 | 0.24011593 | 0.006066906 | 0.4278444 | 0.003750527 | error-prone repair: SOS-response transcriptional repressors (LexA homologs (umuD) | tigr|STM1998 |
| STM2240 | 0.2779207 | 0.00590353 | 0.473031 | 0.002148138 | putative cytoplasmic protein | tigr|STM2240 |
| STM2407 | 0.25137076 | 8.47E-04 | 0.24988502 | 3.36E-05 | putative periplasmic protein (ypeC) | tigr|STM2407 |
| STM2599 | 0.42859784 | 0.00371465 | 0.45409426 | 0.002141577 | Gifsy-1 prophage | tigr|STM2599 |
| STM2611 | 0.0558382 | 0.017144026 | 0.069649704 | 1.49E-07 | Gifsy-1 prophage | tigr|STM2611 |
| STM2801 | 0.48963827 | 0.003843815 | 0.2628839 | 2.53E-04 | putative cytoplasmic protein (ygaC) | tigr|STM2801 |
| STM2829 | 0.25707495 | 0.015274851 | 0.28540742 | 0.002043816 | DNA strand exchange and recombination protein with (recA) | tigr|STM2829 |
| STM3007 | 0.2763739 | 0.005648219 | 0.32442105 | 0.002323769 | putative POT family, peptide transport protein (ygdR) | tigr|STM3007 |
| STM3248 | 0.04960602 | 0.013768029 | 0.36623 | 4.36E-04 | tartronate semialdehyde reductase (TSAR) (garR) | tigr|STM3248 |
| STM3362 | 0.12929411 | 0.019653786 | 0.06711589 | 2.13E-05 | putative periplasmic protein | tigr|STM3362 |
| STM3432 | 0.36229646 | 0.008182923 | 0.4799092 | 0.012927662 | 50S ribosomal subunit protein L29 (rpmC) | tigr|STM3432 |
| STM3437 | 0.31639633 | 0.009825648 | 0.43806115 | 0.003357107 | 50S ribosomal subunit protein L2 (rplB) | tigr|STM3437 |
| STM3438 | 0.42735016 | 0.008721505 | 0.4609047 | 4.29E-04 | 50S ribosomal subunit protein L23 (rplW) | tigr|STM3438 |
| STM3612 | 0.4093345 | 0.005065286 | 0.36995044 | 0.001608172 | ketodeoxygluconokinase (kdgK) | tigr|STM3612 |
| STM3645 | 0.37051162 | 0.019384434 | 0.25434503 | 0.013457329 | putative outer membrane lipoprotein (yiaD) | tigr|STM3645 |
| STM3764 | 0.18117085 | 0.00703069 | 0.21614027 | 0.010616724 | Mg2+ transport protein (mgtC) | tigr|STM3764 |
| STM4033 | 0.10766203 | 0.005635873 | 0.4109749 | 0.00163029 | putative bacterial regulatory protein, merR family | tigr|STM4033 |
| STM4239 | 0.3664815 | 0.003969094 | 0.3760205 | 0.004218783 | putative cytoplasmic protein | tigr|STM4239 |
| STM4240 | 0.4995942 | 0.011368134 | 0.4278014 | 0.008210749 | putative cytoplasmic protein (yjbJ) | tigr|STM4240 |
| STM4265 | 0.30431414 | 0.004534246 | 0.22344881 | 2.54E-04 | transcriptional activator of superoxide response regulon (AraC/XylS (soxS) | tigr|STM4265 |
| STM4284 | 0.42446837 | 0.005994384 | 0.3880425 | 0.013859009 | putative TPR repeat protein (yjcO) | tigr|STM4284 |
| STM4415 | 0.3447196 | 0.003434348 | 0.39201617 | 0.006412169 | fructose-bisphosphatase (fbp) | tigr|STM4415 |
