# Supplementary material for: lac Repressor Is an Antivirulence Factor of Salmonella enterica: Its Role in the Evolution of Virulence in Salmonella
Source: PLoS One. 2009 Jun 4;4(6):e5789. doi: 10.1371/journal.pone.0005789 (PMC2686271; doi:10.1371/journal.pone.0005789)
Supplement: Table S2 — List of genes which are up-regulated in the strain having pTrc99A but not in the strain having pTrc(-LacI) (0.30 MB DOC) [file pone.0005789.s002.doc]

| **Gene** | **Ratio_pTrc99A(cy5) vs. WT (cy3)**  **Table S2: List of genes which are up-regulated in the strain having pTrc99A but not in the strain having pTrc(-LacI)** | ***P*-value** | **Ratio_pTrc99A (cy3) vs. WT (cy5)** | ***P*-value** | **Description** | **accessions** |
| --- | --- | --- | --- | --- | --- | --- |
| PSLT025 | 2.3444784 | 9.45E-06 | 3.0300262 | 3.61E-04 | putative cytoplasmic protein | tigr|PSLT025 |
| STM0110 | 2.517542 | 5.20E-05 | 2.3997827 | 3.48E-04 | 3-isopropylmalate isomerase (dehydratase), subunit with LeuC (leuD) | tigr|STM0110 |
| STM0155 | 3.1675243 | 1.25E-05 | 2.0541806 | 0.007922478 | putative outer membrane protein | tigr|STM0155 |
| STM0462 | 17.884651 | 2.44E-08 | 2.453878 | 1.51E-04 | regulatory protein, P-II 2, for nitrogen assimilation (glnK) | tigr|STM0462 |
| STM0463 | 18.478424 | 3.61E-05 | 3.1220274 | 0.001230252 | putative Amt family, ammonium transport protein (amtB) | tigr|STM0463 |
| STM0512 | 10.70613 | 6.08E-07 | 2.3717196 | 2.98E-04 | putative binding-protein-dependent transport systems inner membrane component (sfbC) | tigr|STM0512 |
| STM0533 | 2.3905122 | 0.00997876 | 3.2847335 | 0.002004745 | phosphoribosylaminoimidazole carboxylase = AIR carboxylase, CO(2)-fixing subunit (purK) | tigr|STM0533 |
| STM0534 | 3.5901406 | 5.28E-04 | 2.6851466 | 0.01860357 | phosphoribosylaminoimidazole carboxylase = AIR carboxylase, catalytic subunit (purE) | tigr|STM0534 |
| STM0596 | 5.1475654 | 1.11E-06 | 2.2467287 | 1.48E-05 | 2,3-dihydroxybenzoate-AMP ligase (entE) | tigr|STM0596 |
| STM0597 | 3.302633 | 7.23E-05 | 2.3808825 | 5.16E-04 | 2,3-dihydro-2,3-dihydroxybenzoate synthetase, isochorismatase (entB) | tigr|STM0597 |
| STM0662 | 4.41768 | 8.70E-08 | 10.117653 | 1.11E-05 | ABC superfamily (atp_bind), glutamate/aspartate transporter (gltL) | tigr|STM0662 |
| STM0663 | 3.4704251 | 1.11E-05 | 4.983233 | 5.41E-04 | ABC superfamily (membrane), glutamate/aspartate transporter (gltK) | tigr|STM0663 |
| STM0664 | 3.0874937 | 2.95E-07 | 4.4876328 | 4.60E-06 | ABC superfamily (membrane), glutamate/aspartate transporter (gltJ) | tigr|STM0664 |
| STM0680 | 9.313563 | 3.06E-06 | 4.467978 | 1.78E-04 | asparagine synthetase B (asnB) | tigr|STM0680 |
| STM0691 | 2.4247289 | 3.37E-04 | 3.1206782 | 4.59E-05 | putative periplasmic protein | tigr|STM0691 |
| STM0778 | 6.2702055 | 1.97E-05 | 2.6571932 | 0.001028285 | putative ABC superfamily (atp_bind), molybdenum transporter (modF) | tigr|STM0778 |
| STM0782 | 2.0795567 | 0.004498783 | 4.2084374 | 0.008850345 | ABC superfamily (membrane), molybdate transporter (modB) | tigr|STM0782 |
| STM0783 | 2.640993 | 6.55E-07 | 4.6512594 | 2.54E-05 | ABC superfamily (atp_bind), molybdate transporter (modC) | tigr|STM0783 |
| STM0827 | 2.2748778 | 7.72E-05 | 3.5728629 | 0.008736293 | paral putative transport protein (ybiO) | tigr|STM0827 |
| STM0828 | 13.8700075 | 2.60E-05 | 6.39083 | 0.004376395 | ABC superfamily (atp_bind), glutamine high-affinity transporter (glnQ) | tigr|STM0828 |
| STM0829 | 4.435619 | 1.21E-05 | 2.1882465 | 9.24E-04 | ABC superfamily (membrane), glutamine high-affinity transporter (glnP) | tigr|STM0829 |
| STM0830 | 3.8389788 | 0.001568654 | 3.0906518 | 0.013636638 | ABC superfamily (bind_prot), glutamine high-affinity transporter (glnH) | tigr|STM0830 |
| STM0834 | 7.348926 | 0.003528077 | 6.1698556 | 0.007285322 | putative Integral membrane protein (ybiP) | tigr|STM0834 |
| STM0848 | 3.255469 | 8.15E-04 | 3.2615829 | 8.46E-04 | putative ATPase components of ABC-type transport system (yliA) | tigr|STM0848 |
| STM0849 | 4.9275475 | 4.72E-04 | 2.6206384 | 0.008083667 | putative ABC transporter periplasmic binding protein (yliB) | tigr|STM0849 |
| STM0872 | 3.9649165 | 2.36E-06 | 2.1224353 | 0.002551834 | glutaredoxin1 redox coenzyme for glutathione-dependent ribonucleotide reductase (grxA) | tigr|STM0872 |
| STM0888 | 5.9947896 | 7.16E-06 | 2.776471 | 9.75E-05 | ABC superfamily (membrane), arginine 3rd transport system (artM) | tigr|STM0888 |
| STM0889 | 5.527287 | 1.85E-05 | 2.677255 | 4.37E-04 | ABC superfamily (membrane), arginine 3rd transport system (artQ) | tigr|STM0889 |
| STM0890 | 4.4153357 | 1.76E-06 | 3.1043127 | 0.003188291 | ABC superfamily (bind_prot), arginine transport system (artI) | tigr|STM0890 |
| STM0933 | 2.6627274 | 3.07E-06 | 2.221598 | 1.43E-04 | putative nucleoside-diphosphate-sugar epimerase (ybjT) | tigr|STM0933 |
| STM0978 | 8.854927 | 1.86E-06 | 2.2828372 | 0.002782656 | 3-enolpyruvylshikimate-5-phosphate synthetase (aroA) | tigr|STM0978 |
| STM1018 | 3.1341538 | 2.16E-07 | 2.494709 | 5.24E-05 | Gifsy-2 prophage | tigr|STM1018 |
| STM1073 | 2.3148055 | 2.88E-05 | 2.302142 | 9.31E-04 | putative efflux (PET) family transporter (yccS) | tigr|STM1073 |
| STM1453 | 2.0536168 | 1.59E-04 | 2.1914651 | 8.10E-04 | endonuclease III DNA glycosylase/apyrimidinic (AP) lyase (nth) | tigr|STM1453 |
| STM1475 | 2.8942475 | 6.77E-05 | 2.4567907 | 0.00116541 | response regulator in two-component regulatory system with (rstA) | tigr|STM1475 |
| STM1512 | 3.011393 | 8.98E-04 | 2.343037 | 0.017250126 | dipeptidyl carboxypeptidase II (dcp) | tigr|STM1512 |
| STM1531 | 2.1305258 | 1.65E-04 | 2.2468064 | 0.003523547 | putative hydrogenase | tigr|STM1531 |
| STM1643 | 3.1816566 | 2.38E-07 | 2.2163048 | 2.42E-05 | putative inner membrane protein | tigr|STM1643 |
| STM1672 | 4.2504787 | 2.74E-06 | 2.5756097 | 0.003824172 | putative cytoplasmic protein | tigr|STM1672 |
| STM1704 | 2.5609763 | 0.003491525 | 2.1064014 | 0.001829056 | putative regulatory protein, deoR family (yciT) | tigr|STM1704 |
| STM1706 | 4.2855964 | 0.007289988 | 2.3211598 | 0.007147709 | putative translation initiation factor SUI1 (yciH) | tigr|STM1706 |
| STM1708 | 4.3274493 | 2.68E-07 | 2.4418566 | 9.38E-05 | putative N-acetylglucosaminyl transferase (yciM) | tigr|STM1708 |
| STM1713 | 5.517405 | 1.01E-06 | 5.882246 | 3.66E-05 | transcriptional regulator for cysteine regulon (LysR familiy) (cysB) | tigr|STM1713 |
| STM1743 | 2.580501 | 3.41E-04 | 2.0860994 | 0.007429842 | ABC superfamily (atp-binding), oligopeptide transport protein (oppD) | tigr|STM1743 |
| STM1751 | 2.3572059 | 1.21E-04 | 2.7046342 | 2.45E-05 | DNA-binding protein HLP-II (HU, BH2, HD, NS) (hns) | tigr|STM1751 |
| STM1776 | 4.9021664 | 1.02E-05 | 2.3784938 | 0.002045926 | peptide chain release factor RF-1 (prfA) | tigr|STM1776 |
| STM1811 | 2.832152 | 1.64E-05 | 2.2307098 | 0.003216906 | putative cytoplasmic protein (ycgN) | tigr|STM1811 |
| STM1816 | 2.9240518 | 2.61E-05 | 2.322032 | 0.003629895 | cell division topological specificity factor, reverses MinC (minE) | tigr|STM1816 |
| STM1836 | 2.7487192 | 4.85E-05 | 2.435852 | 5.23E-05 | putative penicillin-binding protein-3 | tigr|STM1836 |
| STM1839 | 3.3557587 | 1.28E-06 | 2.3071663 | 1.27E-05 | putative periplasmic or exported protein | tigr|STM1839 |
| STM1845 | 2.6866758 | 7.89E-04 | 2.2372293 | 0.012404937 | carboxy-terminal protease for penicillin-binding protein 3 (prc) | tigr|STM1845 |
| STM1862 | 3.9521828 | 0.011373027 | 2.3016095 | 0.005538602 | PhoPQ-activated gene predicted integral membrane protein (pagO) | tigr|STM1862 |
| STM1868A | 31.288092 | 4.56E-07 | 2.1864197 | 0.003387704 | lytic enzyme | tigr|STM1868A |
| STM1883 | 3.4879458 | 0.003799904 | 2.7430568 | 0.016981326 | phosphoribosylglycinamide formyltransferase 2 (purT) | tigr|STM1883 |
| STM1885 | 5.4852247 | 2.34E-04 | 2.1918445 | 0.006335603 | 6-phosphogluconate dehydratase (edd) | tigr|STM1885 |
| STM1915 | 6.592332 | 9.53E-07 | 6.126061 | 7.04E-04 | chemotactic response CheY protein phophatase (cheZ) | tigr|STM1915 |
| STM1916 | 2.7663825 | 1.08E-05 | 5.8418193 | 7.64E-05 | chemotaxis regulator, transmits chemoreceptor signals to flagelllar (cheY) | tigr|STM1916 |
| STM1917 | 2.4163752 | 3.45E-05 | 6.97135 | 1.94E-04 | methyl esterase, response regulator for chemotaxis (cheB) | tigr|STM1917 |
| STM1920 | 2.2660544 | 3.68E-05 | 4.3857536 | 6.49E-04 | purine-binding chemotaxis protein regulation (cheW) | tigr|STM1920 |
| STM1946 | 3.2092175 | 4.68E-05 | 2.1621015 | 0.008475336 | UvrC with UvrAB is a DNA excision (uvrC) | tigr|STM1946 |
| STM1951 | 2.6090624 | 1.08E-06 | 3.2346885 | 1.32E-04 | putative ABC-type polar amino acid transport system (yecC) | tigr|STM1951 |
| STM1953 | 2.266267 | 9.30E-05 | 2.3880777 | 0.002915167 | putative 1-cyclopropane-carboxylate deaminase (yedO) | tigr|STM1953 |
| STM1954 | 4.3409166 | 1.10E-04 | 3.6333945 | 7.23E-04 | putative periplasmic binding transport protein (fliY) | tigr|STM1954 |
| STM1958 | 2.6836352 | 0.003442834 | 4.6727905 | 0.008388908 | N-methylation of lysine residues in flagellin (fliB) | tigr|STM1958 |
| STM1959 | 2.542226 | 0.002753506 | 4.681996 | 0.001316873 | flagellar biosynthesis flagellin, filament structural protein (fliC) | tigr|STM1959 |
| STM1962 | 2.33547 | 5.38E-04 | 5.380803 | 0.002890112 | flagellar biosynthesis possible export chaperone for FliD (fliT) | tigr|STM1962 |
| STM1963 | 3.6847105 | 0.001231944 | 4.7051044 | 0.002635315 | cytoplasmic alpha-amylase (amyA) | tigr|STM1963 |
| STM2009 | 2.388989 | 0.003865387 | 2.1227882 | 0.011568024 | AMP nucleosidase (amn) | tigr|STM2009 |
| STM2073 | 12.868867 | 5.72E-05 | 2.0320954 | 0.010989928 | histidinol phosphate aminotransferase (hisC) | tigr|STM2073 |
| STM2074 | 20.05431 | 4.79E-06 | 2.2457523 | 1.56E-05 | bifunctional: imidazoleglycerol-phosphate dehydratase histidinol-phosphatase (hisB) | tigr|STM2074 |
| STM2076 | 14.048356 | 1.21E-04 | 2.3162146 | 0.001128988 | N-(5-phospho-L-ribosyl-formimino)-5-amino-1-(5-phosphoribosyl)-4-imidazolecarboxamide isomerase (hisA) | tigr|STM2076 |
| STM2078 | 12.71155 | 4.02E-08 | 2.2487254 | 3.26E-04 | bifunctional: phosphoribosyl-AMP cyclohydrolase phosphoribosyl-ATP pyrophosphatase (hisI) | tigr|STM2078 |
| STM2119 | 2.9979036 | 3.02E-04 | 2.6760159 | 0.002658732 | putative inner membrane protein (yegH) | tigr|STM2119 |
| STM2123 | 2.221816 | 2.79E-04 | 2.3626816 | 0.00241222 | putative PAS/PAC domain Diguanylate cyclase/phosphodiesterase domain 1 (yegE) | tigr|STM2123 |
| STM2145 | 4.7450175 | 3.09E-06 | 2.182484 | 0.004876628 | putative regulatory protein, gntR family (yegW) | tigr|STM2145 |
| STM2148 | 5.2491827 | 8.59E-08 | 2.2527654 | 0.001152292 | putative periplasmic protein | tigr|STM2148 |
| STM2161 | 3.0212004 | 1.52E-05 | 2.0389683 | 0.00324436 | putative inner membrane protein | tigr|STM2161 |
| STM2192 | 4.4232388 | 1.27E-05 | 2.0370657 | 1.57E-05 | putative inner membrane protein (yeiB) | tigr|STM2192 |
| STM2194 | 2.300881 | 6.13E-04 | 2.088314 | 0.002996502 | putative esterase (yeiG) | tigr|STM2194 |
| STM2217 | 6.9347186 | 1.03E-06 | 2.3223119 | 3.81E-04 | putative ABC-type dipeptide/oligopeptide/nickel transport systems, permease component (yejB) | tigr|STM2217 |
| STM2220 | 2.6878066 | 9.65E-04 | 2.9452596 | 0.002500938 | putative cytoplasmic protein (yejG) | tigr|STM2220 |
| STM2248 | 3.3199956 | 3.20E-06 | 2.2313125 | 1.66E-04 | heme lyase/disulfide oxidoreductase, cytochrome c-type biogenesis (ccmG) | tigr|STM2248 |
| STM2251 | 2.5339372 | 1.74E-04 | 2.2118592 | 0.008150616 | heme exporter protein C, cytochrome c-type biogenesis (ccmD) | tigr|STM2251 |
| STM2255 | 6.976879 | 5.93E-06 | 3.6356196 | 2.33E-04 | periplasmic nitrate reductase, cytochrome c-type protein (napC) | tigr|STM2255 |
| STM2271 | 5.8401837 | 2.16E-06 | 2.0400953 | 7.61E-04 | sensory histidine kinase in two-component regulatory system (rcsC) | tigr|STM2271 |
| STM2298 | 6.371599 | 4.27E-06 | 2.473605 | 0.002827565 | putative glycosyl transferase (pmrF) | tigr|STM2298 |
| STM2299 | 16.743738 | 2.92E-07 | 2.32324 | 0.002113854 | paral putative transformylase (yfbG) | tigr|STM2299 |
| STM2300 | 10.679334 | 1.21E-06 | 2.3573096 | 0.001216148 | putative cytoplasmic protein | tigr|STM2300 |
| STM2314 | 2.819615 | 0.002307326 | 5.296396 | 0.004557867 | putative chemotaxis signal transduction protein | tigr|STM2314 |
| STM2351 | 3.2030103 | 2.12E-05 | 6.14956 | 6.65E-04 | ABC superfamily (atp_bind), histidine and lysine/arginine/ornithine transport (hisP) | tigr|STM2351 |
| STM2352 | 3.2808378 | 2.21E-06 | 4.354695 | 7.35E-04 | ABC superfamily (membrane),histidine and lysine/arginine/ornithine transport protein (hisM) | tigr|STM2352 |
| STM2353 | 3.8042316 | 6.49E-07 | 4.8510294 | 4.82E-05 | ABC superfamily (membrane),histidine and lysine/arginine/ornithine transport system (hisQ) | tigr|STM2353 |
| STM2362 | 3.4525754 | 4.71E-07 | 2.3409019 | 1.15E-04 | amidophosphoribosyltransferase (PRPP amidotransferase) (purF) | tigr|STM2362 |
| STM2363 | 2.8676043 | 3.99E-04 | 2.2775931 | 0.01546824 | membrane protein required for colicin V production (cvpA) | tigr|STM2363 |
| STM2381 | 5.3532143 | 2.23E-07 | 2.0439632 | 4.14E-04 | putative cytoplasmic protein (yfcM) | tigr|STM2381 |
| STM2382 | 4.389925 | 2.22E-05 | 2.0972958 | 0.002128213 | putative permease (yfcA) | tigr|STM2382 |
| STM2441 | 7.196826 | 3.20E-04 | 7.996724 | 5.05E-06 | ABC superfamily (atp_bind), sulfate permease A protein (cysA) | tigr|STM2441 |
| STM2442 | 15.124785 | 7.63E-09 | 24.24672 | 6.12E-06 | ABC superfamily (membrane), thiosulfate permease W protein (cysW) | tigr|STM2442 |
| STM2444 | 8.402963 | 5.08E-07 | 8.950881 | 1.42E-04 | ABC superfamily (bind_prot), thiosulfate transport protein (cysP) | tigr|STM2444 |
| STM2487 | 10.475951 | 2.94E-06 | 4.927598 | 1.07E-04 | phosphoribosylaminoimidazole-succinocarboxamide synthetase (SAICAR synthetase) (purC) | tigr|STM2487 |
| STM2499 | 7.1289086 | 1.84E-07 | 3.3203764 | 0.001073447 | phosphoribosylaminoimidazole synthetase (AIR synthetase) (purM) | tigr|STM2499 |
| STM2500 | 4.1366534 | 6.59E-06 | 2.3353033 | 0.005834917 | polyphosphate kinase, component of RNA degradosome (purN) | tigr|STM2500 |
| STM2540 | 3.891341 | 6.18E-04 | 3.336801 | 0.001674171 | co-chaperone protein Hsc20, believed to be involved (hscB) | tigr|STM2540 |
| STM2541 | 4.565194 | 2.38E-07 | 3.0709977 | 1.84E-04 | putative regulator (yfhF) | tigr|STM2541 |
| STM2542 | 4.122402 | 5.42E-06 | 3.0511005 | 0.001010536 | NifU homologs involved in Fe-S cluster formation (nifU) | tigr|STM2542 |
| STM2543 | 3.0957322 | 2.65E-05 | 3.0082884 | 8.42E-05 | putative aminotransferase class-V (nifS) | tigr|STM2543 |
| STM2554 | 2.1480367 | 0.001383748 | 2.3938427 | 0.014693798 | putative MFS family transport protein (hcaT) | tigr|STM2554 |
| STM2622 | 2.9897482 | 2.65E-05 | 2.4608946 | 0.002166231 | Gifsy-1 prophage | tigr|STM2622 |
| STM2646 | 35.75821 | 7.39E-07 | 2.0543423 | 3.01E-04 | putative formate acetyltransferase (yfiD) | tigr|STM2646 |
| STM2653 | 3.3142056 | 5.91E-07 | 2.6271167 | 3.80E-05 | putative outer membrane lipoprotein (yfiM) | tigr|STM2653 |
| STM2774 | 9.615198 | 4.21E-05 | 2.3353786 | 0.005162384 | putative ATP binding cassette (ABC) transporter (iroC) | tigr|STM2774 |
| STM2808 | 5.4935737 | 5.56E-04 | 2.6916213 | 0.017837692 | ribonucleoside-diphosphate reductase 2, beta subunit (nrdF) | tigr|STM2808 |
| STM2810 | 4.965753 | 1.38E-05 | 2.5881572 | 0.002376037 | ABC superfamily (membrane), glycine/betaine/proline transport protein (proW) | tigr|STM2810 |
| STM2811 | 8.775655 | 2.89E-06 | 3.06007 | 1.62E-04 | ABC superfamily (bind_prot), glycine/betaine/proline transport protein (proX) | tigr|STM2811 |
| STM2866 | 3.810172 | 4.17E-04 | 2.2730284 | 0.019108083 | transcriptional regulator (sprB) | tigr|STM2866 |
| STM2871 | 3.0001373 | 3.02E-04 | 4.208591 | 1.42E-04 | cell invasion protein lipoprotein, may link inner (prgK) | tigr|STM2871 |
| STM2890 | 2.1425865 | 3.52E-04 | 2.986225 | 0.001194063 | surface presentation of antigens secretory proteins (spaP) | tigr|STM2890 |
| STM2891 | 2.253795 | 1.05E-04 | 3.2233114 | 0.002303247 | surface presentation of antigens secretory proteins (spaO) | tigr|STM2891 |
| STM2892 | 2.2737749 | 0.003006341 | 3.015634 | 0.016129412 | surface presentation of antigens secretory proteins (invJ) | tigr|STM2892 |
| STM2932 | 10.812862 | 8.98E-05 | 5.5407333 | 5.07E-04 | putative inner membrane protein (ygbE) | tigr|STM2932 |
| STM2933 | 7.8470993 | 6.62E-06 | 8.051956 | 8.02E-04 | adenosine 5-phosphosulfate kinase (cysC) | tigr|STM2933 |
| STM2946 | 19.53864 | 5.48E-09 | 15.279612 | 5.06E-06 | 3-phosphoadenosine 5-phosphosulfate (PAPS) reductase (cysH) | tigr|STM2946 |
| STM2947 | 15.085768 | 7.31E-06 | 18.053885 | 3.78E-04 | sulfite reductase, alpha subunit, NADPH dependent (cysI) | tigr|STM2947 |
| STM2948 | 3.318249 | 8.44E-05 | 10.628444 | 7.49E-05 | sulfite reductase, beta (flavoprotein) subunit (cysJ) | tigr|STM2948 |
| STM2992 | 2.2432942 | 4.50E-05 | 3.105424 | 0.008362757 | N-alpha-acetylglutamate synthase (amino-acid acetyltransferase) (argA) | tigr|STM2992 |
| STM3151 | 4.4637227 | 2.55E-06 | 4.833077 | 2.19E-05 | putative cytoplasmic protein (yghW) | tigr|STM3151 |
| STM3216 | 2.621284 | 0.001283198 | 3.0583558 | 0.011094047 | putative methyl-accepting chemotaxis protein | tigr|STM3216 |
| STM3330 | 6.960477 | 5.94E-08 | 2.5260236 | 1.15E-05 | glutamate synthase, large subunit (gltB) | tigr|STM3330 |
| STM3468 | 8.680519 | 2.11E-06 | 2.3095884 | 0.001819346 | acetylornithine transaminase (NAcOATase and DapATase) (argD) | tigr|STM3468 |
| STM3476 | 14.269875 | 2.12E-07 | 4.011558 | 0.001123184 | FNT family, nitrite transport protein (nirC) | tigr|STM3476 |
| STM3477 | 8.759771 | 3.10E-04 | 2.1472213 | 0.01797997 | siroheme synthase, catalyses four separate reactions that (cysG) | tigr|STM3477 |
| STM3576 | 3.219594 | 8.81E-04 | 2.5341475 | 0.010661718 | P-type ATPase family, Pb/Cd/Zn/Hg transporting ATPase (zntA) | tigr|STM3576 |
| STM3577 | 2.9032497 | 1.53E-04 | 6.53653 | 1.95E-04 | methyl-accepting transmembrane citrate/phenol chemoreceptor (tcp) | tigr|STM3577 |
| STM3579 | 6.8335423 | 7.05E-05 | 3.8241637 | 8.06E-04 | putative integral membrane protein (yhhQ) | tigr|STM3579 |
| STM3745 | 2.162309 | 0.003638661 | 2.1741924 | 0.013369555 | putative cytoplasmic protein | tigr|STM3745 |
| STM3877 | 33.366684 | 7.35E-05 | 2.731713 | 0.004025457 | asparagine synthetase A (asnA) | tigr|STM3877 |
| STM3900 | 3.8955538 | 3.12E-05 | 2.5015955 | 0.002964782 | ilvGEDA operon leader peptide (ilvL) | tigr|STM3900 |
| STM4002 | 5.3369904 | 4.75E-04 | 2.0669415 | 0.004599611 | putative cytoplasmic protein | tigr|STM4002 |
| STM4005 | 6.431673 | 5.56E-04 | 3.8012397 | 0.007153039 | response regulator in two-component regulatory system with (glnG) | tigr|STM4005 |
| STM4006 | 4.39832 | 1.23E-06 | 2.9609594 | 3.53E-05 | sensory kinase (phosphatase) in two-component regulatory system (glnL) | tigr|STM4006 |
| STM4007 | 18.247637 | 2.37E-07 | 3.1430864 | 8.64E-04 | glutamine synthetase (glnA) | tigr|STM4007 |
| STM4123 | 6.014869 | 3.27E-06 | 2.992284 | 4.22E-04 | argininosuccinate lyase (argH) | tigr|STM4123 |
| STM4161 | 4.289326 | 6.12E-06 | 2.747556 | 0.005975221 | putative involved in thiamine biosynthesis | tigr|STM4161 |
| STM4162 | 4.4801674 | 2.13E-04 | 2.3468254 | 0.011531252 | catalyzes the adenylation of thisS as part (thiF) | tigr|STM4162 |
| STM4176 | 3.5944228 | 3.08E-05 | 2.5978522 | 2.30E-04 | bifunctional: phosphoribosylaminoimidazolecarboxamide formyltransferase IMP cyclohydrolase (purH) | tigr|STM4176 |
| STM4290 | 3.5310545 | 4.58E-04 | 2.3064084 | 0.003571302 | MFS family, low-affinity proline transporter (proline permease (proP) | tigr|STM4290 |
| STM4469 | 8.53042 | 1.56E-08 | 2.5714004 | 8.54E-05 | ornithine carbamoyltransferase 1 (argI) | tigr|STM4469 |
