# Supplementary material for: lac Repressor Is an Antivirulence Factor of Salmonella enterica: Its Role in the Evolution of Virulence in Salmonella
Source: PLoS One. 2009 Jun 4;4(6):e5789. doi: 10.1371/journal.pone.0005789 (PMC2686271; doi:10.1371/journal.pone.0005789)
Supplement: Table S3 — Plasmid constructs used in this study (0.03 MB DOC) [file pone.0005789.s003.doc]

**Table S3. Plasmid constructs used in this study**

| **Plasmid*** | **Relevant characteristics** | **Reference** |
| --- | --- | --- |
| pTrc99A | A plasmid with colE1 replicon expressing *lac* repressor | [1] |
| pTrc(-LacI) | pTrc99A without *lac* repressor | This study |
| pBR322 | A plasmid with colE1 replicon; does not have *lacI* | [2] |
| pBR322(+LacI) | pBR322 expressing *lac* repressor | This study |
| pJC1[LacI(Gly^60+3^)] | pJC1 plasmid expressing a mutant LacI that cannot bind to the operator sequence | [3] |

* All these plasmids were individually transformed into the WT *S*. *enterica* to get corresponding strains.

**References:**

1. Amann E, Ochs B, Abel KJ (1988) Tightly regulated tac promoter vectors useful for

the expression of unfused and fused proteins in Escherichia coli. Gene 69: 301-315.

2. Bolivar F, Rodriguez RL, Greene PJ, Betlach MC, Heyneker HL, et al. (1977)

Construction and characterization of new cloning vehicles. II. A multipurpose cloning

system. Gene 2: 95-113.

3. Falcon CM, Matthews KS (1999) Glycine insertion in the hinge region of lactose

repressor protein alters DNA binding. J Biol Chem 274: 30849-30857.
