# Supplementary material for: lac Repressor Is an Antivirulence Factor of Salmonella enterica: Its Role in the Evolution of Virulence in Salmonella
Source: PLoS One. 2009 Jun 4;4(6):e5789. doi: 10.1371/journal.pone.0005789 (PMC2686271; doi:10.1371/journal.pone.0005789)
Supplement: Table S4 — Primers used in this study (0.03 MB DOC) [file pone.0005789.s004.doc]

Table S4. Primers* used in this study

| cloning of *lacI* in pBR322 | Forward: tgcggatccgaattcaagcggcatgcatttacgttg  Reverse: tgcaagcttcgcgctaactcacattaa |
| --- | --- |
| cloning of *lacI(1-60)* in pTrc(-LacI) | Forward: tgcggatccgaattcaagcggcatgcatttacgttg  Reverse: agtcaagctttcactgtttgcccgccagttgttg |
| cloning of *lacI(61-360)* in  pTrc(-LacI) | Forward: agtcccatgggctcgttgctgattggcgttgcc  Reverse: agtcggatcctcactgcccgctttccagtcg |
| *lacI* RT-PCR | Forward: attacattcccaaccgcgt  Reverse: ttctaccatcgacaccacca |
| *spiC* RT-PCR | Forward: ggattcatgctggcagtttt  Reverse: cataggcaagacaaggcttag |
| *sseB* RT-PCR | Forward: ccgaagggtatggtgttttg  Reverse: tggttttagcatcccctttg |
| *ssaK* RT-PCR | Forward: aaaatcatccgagacgccta  Reverse: tgatcgaccaatgaacgaaa |
| *rpoD* RT-PCR | Forward: gtggcttgcaattccttgat  Reverse: agcatctggcgagaaatacg |
| *phoP* RT-PCR | Forward: gaaggctggcaggataaagt  Reverse: ccgtgagtttgatgacctct |
| *16S rRNA* RT-PCR | Forward: gatcatggctcagattgaacgctggcgg  Reverse: caccgctacacctggaattctacccccctc |

* All nucleotide sequences are given in 5' to 3' direction
