# Supplementary figures and images for: lac Repressor Is an Antivirulence Factor of Salmonella enterica: Its Role in the Evolution of Virulence in Salmonella
Source: PLoS One. 2009 Jun 4;4(6):e5789. doi: 10.1371/journal.pone.0005789 (PMC2686271; doi:10.1371/journal.pone.0005789)

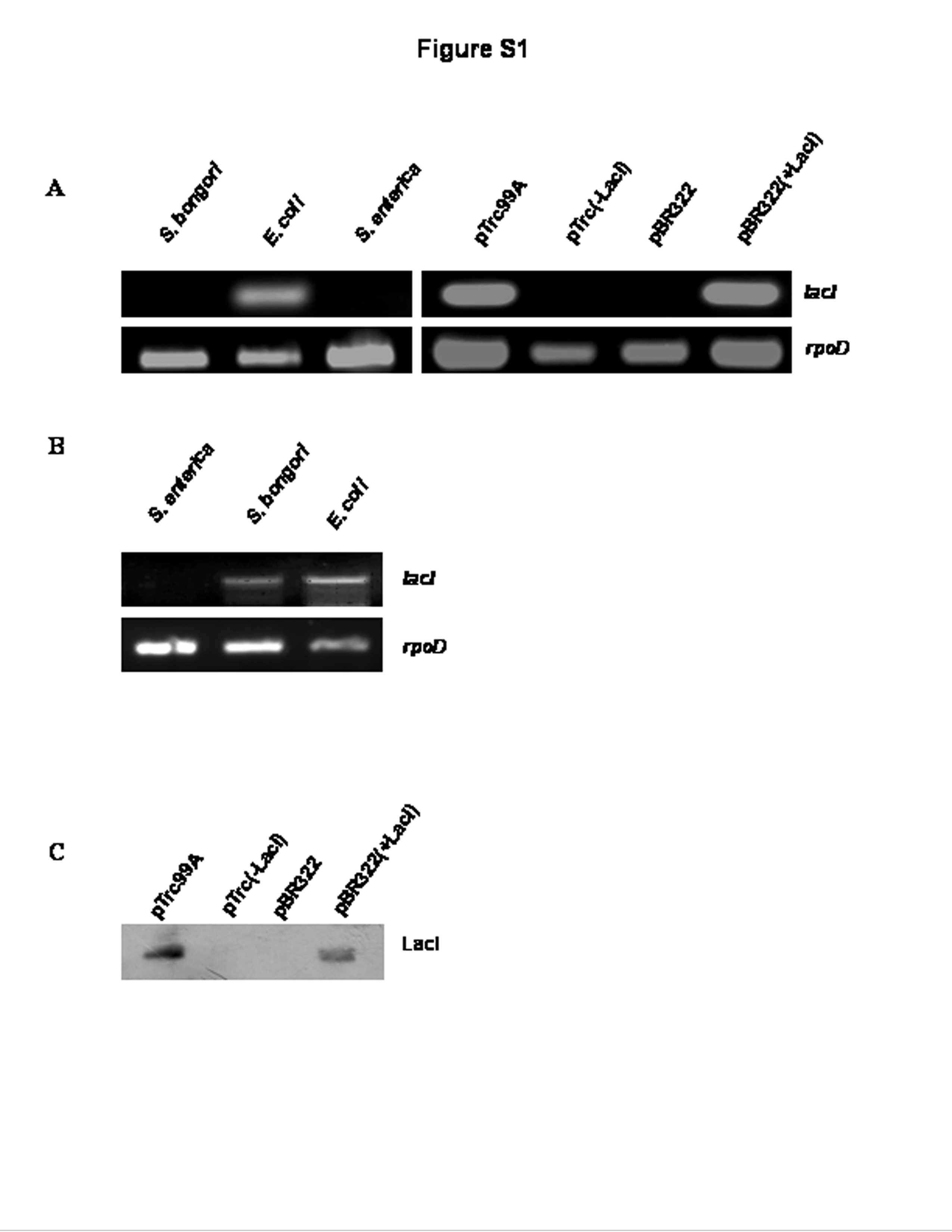

Supplement: Figure S1 — Analysis of expression of LacI. (A) RT-PCR analysis of lacI expression in different strains of Salmonella and E. coli. RNA isolated from overnight culture of bacteria was treated with RNase-free DNase and reverse transcribed. cDNA thus generated was used for PCR amplification. (B) PCR amplification of lacI from the genomic DNA of bacteria. In both (A) and (B), PCR was done using lacI-specific primers. rpoD was used as internal control. (C) Western blot analysis of LacI expression in different strains of Salmonella. Equal number of bacteria from overnight culture was lysed and the lysate was used for Western blot analysis. LacI antibody was from Abcam. (1.24 MB TIF) [file pone.0005789.s006.tif]
